# Enhanced Motor Learning Following Task-Concurrent Dual Transcranial Direct Current Stimulation

**DOI:** 10.1371/journal.pone.0085693

**Published:** 2013-12-23

**Authors:** Sophia Karok, Alice G. Witney

**Affiliations:** Department of Physiology, School of Medicine, Biomedical Sciences Institute, Trinity College Dublin, Dublin, Ireland; University of California, Merced, United States of America

## Abstract

**Objective:**

Transcranial direct current stimulation (tDCS) of the primary motor cortex (M1) has beneficial effects on motor performance and motor learning in healthy subjects and is emerging as a promising tool for motor neurorehabilitation. Applying tDCS concurrently with a motor task has recently been found to be more effective than applying stimulation before the motor task. This study extends this finding to examine whether such task-concurrent stimulation further enhances motor learning on a dual M1 montage.

**Method:**

Twenty healthy, right-handed subjects received anodal tDCS to the right M1, dual tDCS (anodal current over right M1 and cathodal over left M1) and sham tDCS in a repeated-measures design. Stimulation was applied for 10 mins at 1.5 mA during an explicit motor learning task. Response times (RT) and accuracy were measured at baseline, during, directly after and 15 mins after stimulation. Motor cortical excitability was recorded from both hemispheres before and after stimulation using single-pulse transcranial magnetic stimulation.

**Results:**

Task-concurrent stimulation with a dual M1 montage significantly reduced RTs by 23% as early as with the onset of stimulation (p<0.01) with this effect increasing to 30% at the final measurement. Polarity-specific changes in cortical excitability were observed with MEPs significantly reduced by 12% in the left M1 and increased by 69% in the right M1.

**Conclusion:**

Performance improvement occurred earliest in the dual M1 condition with a stable and lasting effect. Unilateral anodal stimulation resulted only in trendwise improvement when compared to sham. Therefore, task-concurrent dual M1 stimulation is most suited for obtaining the desired neuromodulatory effects of tDCS in explicit motor learning.

## Introduction

Transcranial direct current stimulation (tDCS) is a non-invasive stimulation technique that induces robust excitability changes in the human motor cortex [1,2]. Recently, it has attracted interest as a promising tool for rehabilitating the loss of motor function after stroke [3]. TDCS differs from other non-invasive brain stimulation techniques such as transcranial magnetic stimulation (TMS) in that it applies low-amplitude direct currents (2 mA or less) over the stimulated cortex and modulates spontaneous neural activity as opposed to inducing neuronal firing by supra-threshold neuronal membrane potential [4]. Therefore, tDCS is a neuromodulatory intervention, which allows its application to be simpler, less painful and easier to sham [5]. It is now being re-explored, with different, optimised parameters of stimulation, including intensity and duration of stimulation as well as electrode montage.

TDCS of the primary motor cortex (M1) is an important target for motor learning and rehabilitation [6], as M1 is involved in the early consolidation of motor skills [7]. Therefore, a tDCS-driven enhancement of cortical excitability facilitates neuronal recruitment of this area for motor learning. The underlying mechanisms could be a direct effect on M1 or an indirect effect on motor regions connected with M1; the premotor area, the supplementary motor area and the contralateral M1 [8-10]. The output of M1 can be objectively measured in the form of motor evoked potentials (MEPs) to elucidate changes in corticospinal excitability. Indeed, Nitsche & Paulus, with their influential paper, reintroduced tDCS by demonstrating the immediate and polarity-specific effects of tDCS on motor excitability [2]. The electrode montage used in this study, with the active electrode over the hand area of the motor cortex and the reference electrode over the contralateral orbit, is regarded as one of the most effective for motor learning applications.

However, recent research demonstrated that bilaterally stimulating both motor cortices simultaneously was more effective at improving performance at a finger-sequencing task than the conventional unilateral montage [11]. Vines et al. placed the cathode over the M1 ipsilateral to the motor performing hand. It was suggested that the excitatory effects of the contralateral M1 receiving the anodal current is further augmented by simultaneously being released from inhibitory projections coming from its counterpart in the other hemisphere, as achieved by the cathodal current. The differential effects from this dual motor cortex stimulation seem to be mediated by synergistic bihemispheric network modulations [12-14]. These interhemispheric transmissions, that are predominantly inhibitory, are imbalanced in persons with unilateral lesions. Dual tDCS has been particularly successful as a neurorehabilitative measure to increase motor skill recovery [15-18]. It not only facilitates neural activity in the lesioned hemisphere, but also rebalances the level of inhibition the motor cortices exert on each other [19]. Since motor learning is a key component for stroke recovery, enhancing it is crucial for treatment success.

However, despite the clinical effectiveness of dual M1 stimulation, there are neuronal compensation mechanisms in the lesioned brain as well as post-injury differences across the patient population [20]. An understanding of the behavioural and neurophysiological consequences of dual M1 stimulation in the healthy brain thus benefits development of appropriate protocols. A study that directly compared dual tDCS to unilateral M1 stimulation reported an equal benefit of both stimulation protocols on a finger-sequencing task with the dominant hand relative to sham stimulation [21]. There was a non-significant trend for a higher effect size in the dual condition and, in another study looking at non-dominant hand performance, Williams et al. [22] found that stimulating both M1s simultaneously considerably enhanced performance of healthy subjects on the Jebsen-Taylor Hand Function Task (JTT). 

Since tDCS modulates and does not induce neuronal firing, performing a behavioural task concurrently would enhance the stimulation-induced differences in cortical excitability. In line with this, Stagg et al. [23] reported a polarity-specific change in performance on an explicit 11-digit finger-sequencing coordination task when stimulation was applied concurrently, but not when it was applied before the task. Another group published similar findings in that anodal tDCS to the left M1 improved performance on a serial reaction time task when applied during the task [24], but not when the task is performed after a period of stimulation [25]. The same has been reported for a different type of task: Anodal tDCS was found to enhance motor performance on a stop-signal task when applied during the task, whereas applying the stimulation before the task was more comparable to the results from the sham condition [26]. 

 Therefore, the present study aimed to establish how the dual M1 electrode montage benefits from task-concurrent stimulation. Anodal tDCS to the right M1, dual M1 stimulation and sham stimulation were applied during an explicit motor learning task and performance was compared between the conditions before, during, immediately after and 15 mins after the end of stimulation. We tested the hypothesis that dual M1 stimulation would improve finger-sequencing coordination more than unilateral anodal stimulation and compared both against the sham stimulation condition. Motor cortical excitability was measured before and after the intervention to elucidate possible differences between the conditions and we examined whether it is possible to achieve bidirectional simultaneous modulation of cortical excitability in the dual stimulation condition. 

## Materials and Methods

### Ethics statement

 Subjects provided written informed consent and the experimental protocol was performed in accordance with the Declaration of Helsinki and approved by the Faculty of Health Sciences Research Ethics Committee, Trinity College Dublin, Ireland.

### Subjects

Twenty healthy young adults (12 females; mean age 25.6 years ± 4.5 SD) participated in this study. They showed no signs of any medical or neurological disease or intake of any CNS-active medication as evaluated by a medical questionnaire that was examined by a health practitioner. All were right handed, as determined by the Edinburgh Handedness Inventory [27]. 

### Transcranial direct current stimulation

 TDCS was delivered through two saline-soaked, sponge electrodes using a battery-driven, constant-current stimulator (Magstim Company Limited, Whitland, Wales, UK). The electrodes were held in place with use of a MindCap (NewRonika, Italy).

In all conditions, the anode (25 cm^2^) was fixed over the right M1, corresponding to position C4 of the 10-20 EEG system, centred over the ‘hot-spot’ of the resting first dorsal interosseus (FDI) hand muscle as determined by single-pulse TMS. In the dual condition, the cathode (25 cm^2^) was placed over the FDI hand area of the left M1, corresponding to position C3, also determined by single-pulse TMS. In the anodal condition, the cathode (35 cm^2^) was fixed as a reference electrode contralaterally at the fronto-orbital region or F3 of the 10-20 EEG system. This position for the return electrode has been shown to be functionally ineffective in experimental designs [28]{Nitsche, 2007 #22}. For the sham condition, the electrode montage was pseudo-randomly assigned to be the same as in either the anodal or dual condition.

Current intensity was 1.5 mA (current density of 0.06 mA/cm^2^) and applied for 10 mins. For sham stimulation, current flow increased gradually over a 5s interval reaching the designated 1.5 mA to mimic the initial sensation of real tDCS and the stimulation was then ramped down after 10s and decreased gradually over a 5s interval, so that a conditioning effect on cortical excitability was not induced.

### Measurement of cortical excitability

To detect changes of corticospinal excitability, motor-evoked potentials (MEPs) of the resting FDI were recorded after stimulation of their motor-cortical representational fields by single-pulse TMS. Electromyography (EMG) signals were amplified, band-pass filtered (10 to 50 Hz) and sampled at 1,000 Hz, using conductive adhesive Ag/AgCl electrodes (Tyco Healthcare, Mansfield, UK) in a belly tendon montage. EMGs were recorded through an Octal BioAmp (AD Instruments, Oxford, UK). The peak-to-peak amplitudes of the MEPs were detected by an AD-Instrument custom-written script (LabChart v.7, AD Instruments, Oxford, UK).

TMS was applied using a Magstim Rapid stimulator (Magstim Company Limited, Whitland, Wales, UK) connected to a figure of eight coil (70mm). The coil was positioned tangentially on the scalp with the handle pointing backward at an angle of 45° to the midline. It was placed over the contralateral motor cortex at the optimal site for stimulating the FDI. This optimum coil position (‘hot-spot’) was deﬁned as the position where TMS consistently resulted in the largest mean MEP amplitude. A tight-fitting Lycra cap was placed on the subjects’ head to mark the optimal coil position for TMS. Once placed, the cap and head were marked such as to be able to fit it in the same place when re-fitting the cap after tDCS. 

The resting motor threshold (RMT) was defined as the minimum TMS intensity, which achieved peak-to-peak MEP amplitude of 50-100µV in the resting FDI muscle in 3 out of 6 stimulations. Twenty MEPs (in intervals of 3.5 s) at an intensity that was 120% RMT were acquired from each hemisphere at the three time points PRE, POST1 and POST2 (see section 2.2). The hemisphere that was tested first was randomised and counterbalanced for every measurement.

### Explicit motor learning task

The experiment was run in Psychopy, an open-source experimental-control software package [29]. Subjects were seated in front of a desktop computer with a monitor at eye level and placed the four fingers of their left (non-dominant) hand on the keyboard in front of them. The non-dominant hand was used since it is less trained and more susceptible to performance improvements. The response keys (‘G’, ‘U’, ‘I’, ‘L’) were singled out with all nearby keys removed and labelled ‘6’, ‘7’, ‘8’, ‘9’. This was done to encourage natural finger positioning. 

Instructions for a single trial were to memorise the six-sequence numerical pattern made up of the numbers 6-9 and repeat the movements of this key sequence six times as quickly and as accurately as possible with the corresponding buttons on the keyboard with their left hand (6 = little finger, 7= ring finger, 8 = middle finger, 9 = index finger). During memorising, the four consecutive boxes labelled ‘6’, ‘7’, ‘8’, ‘9’ were always displayed at the centre of the screen and these were highlighted with an asterisk one at a time for 500 ms. Subjects were shown the pattern twice before a ‘Go’ command signalled them to start the finger response. Reaction time (RT) was recorded from the appearance of the ‘go’ signal and RT was recorded for every individual button press. After the sixth repetition of the pattern, the instructions “Hit ‘S’ for next sequence” appear on the screen and the subject would initiate the next trial. There were 12 trials in total and the total duration of the task was 6 min on average. 

All responses were recorded during a trial and so it was any 36 button presses of either 6, 7, 8 or 9 that would be counted before initiating the next trial, as opposed to 36 ‘correct’ button presses. In order to maintain fluency of the finger movements, subjects were instructed to keep going with the pattern, even if a number was ‘missed’ rather than start from the beginning of the sequence. 

At the start of every experimental session, subjects carried out two trials (corresponding to two sequences) as practice trials, but these were the only practice trials in the entire session such as to not miss potential immediate effects following tDCS intervention.

The sequences were chosen to be of varying difficulty, in terms of use of the less dextrous little and ring finger (e.g. 679769 vs. 879698) and in terms of ease of memorising the sequence (e.g. 879698 vs. 986987). Sequences never included consecutives of the same numbers and an effort was made to equalise the ratio of digit presses across sequences. The motor task always used the same 12 sequences, however, there were 3 versions of the task, in which the order of the sequences was randomised. Subjects would use one version of the task per experimental session, so that each subject used each version once, this was pseudo-randomised and counterbalanced.

### Experimental design

A sham-controlled, repeated-measures design was carried out. Each subject participated in three testing sessions, during which they received anodal, dual or sham tDCS. The order of condition was randomised and best care was taken to counterbalance carefully, but the condition order combinations DAS and SAD (where D = “dual”, A = ”anodal” and S = ”sham”) were applied four times whereas all other combinations (i.e. ADS, ASD, DSA and SDA) were applied three times. The order of condition was into account in statistical analyses. All stimulation sessions were separated by at least 1 week.

**Figure 1:**
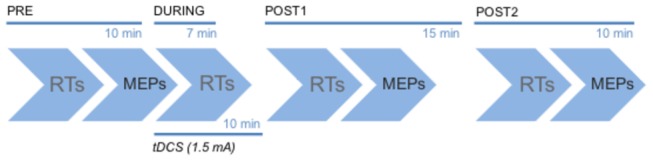
The experimental protocol for every session. The measurements of reaction times the motor task (RT) and the measurements of motor cortical excitability (MEP) were acquired following the same systematic order as shown in this diagram. There were a total of four measurements of motor performance and three measurements of motor cortical excitability.

Motor cortical excitability and performance on the explicit motor learning task were measured at several time points (Figure *1*). The baseline measures were taken for both (PRE), followed by another application of the motor task that started at the same time as the 10-min direct current stimulation (DURING). Due to the opportunity to take short breaks between numerical sequences in the motor task, the stimulation duration was slightly overshot to ensure that the task was at no time performed after a period of stimulation. The motor task was applied again immediately after stimulation (POST1) and a fourth time (POST2), which was applied on average about 15 mins after the end of the stimulation. There were three measurements of motor cortical excitability in total, which were baseline (PRE), POST1 (on average, 8 mins after tDCS) and POST2 (on average, 20 mins after tDCS).

At the end of every experimental session, the subjects completed a visual analogue scale to rate (from 1-7) their current level of fatigue, concentration and perceived pain during the tDCS stimulation.

### Data analyses

 Motor performance on the explicit motor learning task was quantified by the average reaction times (RTs) of every button press and by the per cent accuracy. The standard deviation of RTs for every block was calculated as an index of variability of RTs. For statistical analyses, the mean RTs and the mean accuracy were analysed for every block and compared against each other. Normality was tested using the Shapiro-Wilk test of normality. Where motor parameters were not normally distributed, they were found to be normally distributed in log-space and were transformed logarithmically before statistical analyses. The MEP data were transformed using square root transformation to obtain normal distribution before statistical analyses. The data shown in the tables and figures are untransformed values. Where the assumption of sphericity was not met, the Greenhouse-Geisser correction was applied. All data are reported as mean ± standard deviation (SD).

Statistical analyses were performed separately for each via a two-way repeated-measures (condition: anodal, dual, sham; condition x time points: PRE, DURING, POST1, POST2) analysis of variance (ANOVA). Due to the considerable practice effects of the motor task, the order of conditions from every subject will be entered as a covariate in an additional analysis of covariance (ANCOVA) for the mean RTs.

 For the changes in motor cortical excitability, the MEP amplitudes for each subject were averaged for every block and a two-way repeated-measures ANOVA (condition: anodal, dual, sham; condition x time points: PRE, POST1, POST2) was conducted for each hemisphere separately (left, right). All statistical analyses were performed using SPSS v.20 (SPSS Inc., Chicago, IL, USA). 

## Results

### Behavioural data: performance on the motor task

**Figure 2:**
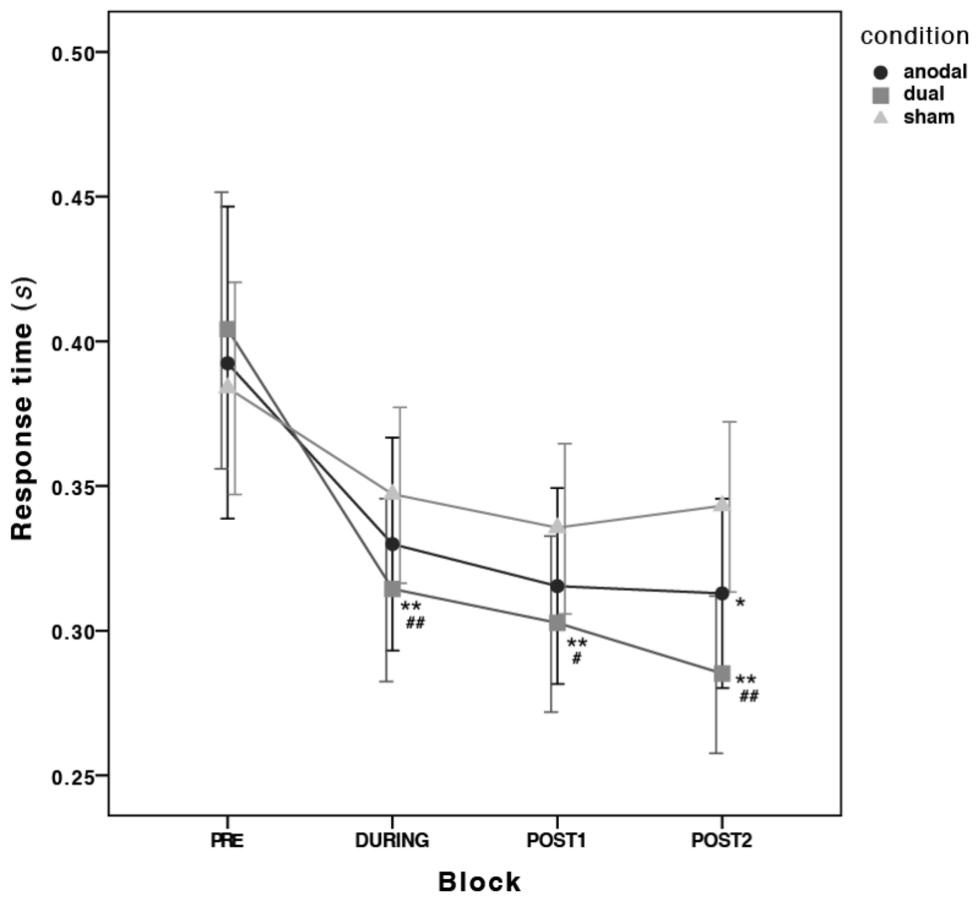
Evolution of reaction times at baseline (PRE), during tDCS (DURING) and at post-intervention (POST1 and POST2) for each active stimulation condition (anodal, dual) compared to sham. * = p<0.05 and ** = p<0.01 from the ANOVA; # = p<0.05 and ## = p<0.01 from the ANCOVA; Error bars are 95% confidence intervals (CI) calculated on the transformed data and transformed back to the original scale.

**Figure 3:**
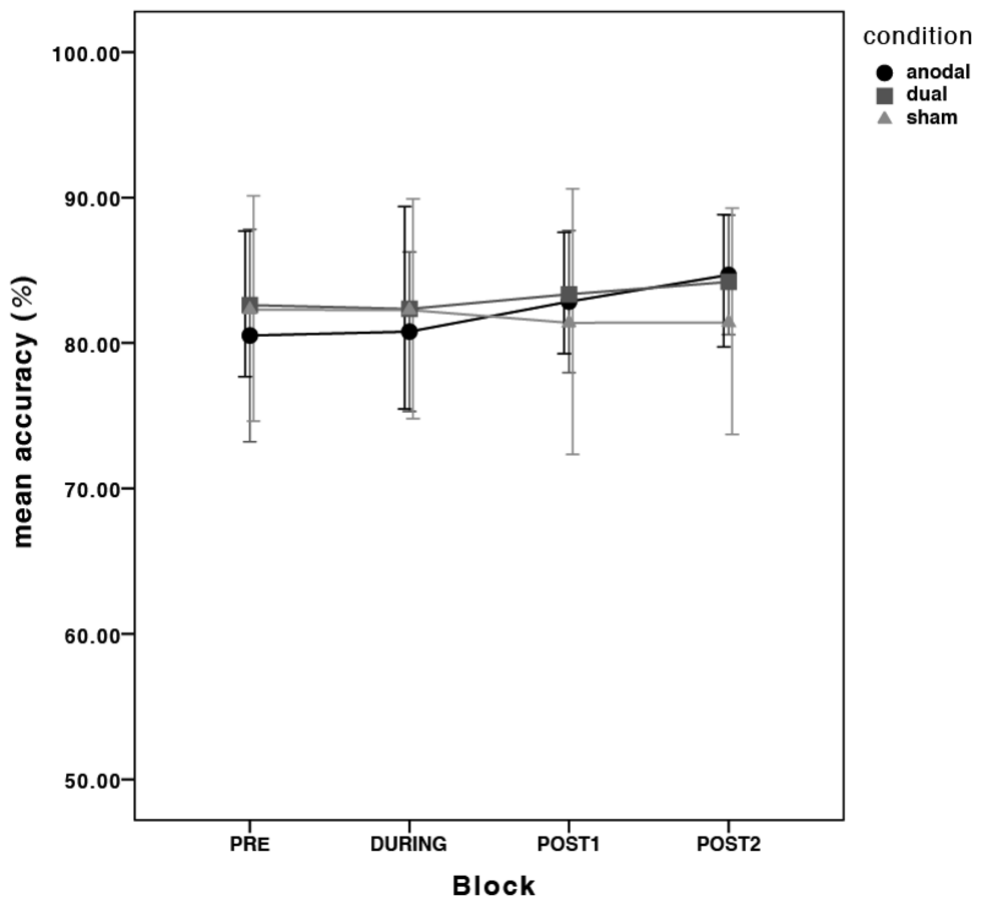
There was very little fluctuation of accuracy across the time points and across stimulation conditions. Error bars are 95% confidence intervals (CI) calculated on the transformed data and transformed back to the original scale.

 All 20 subjects completed the 3 experimental sessions. The motor performance data is depicted in Figure *2* and Figure *3* and the mean RTs and mean accuracy is shown in Table 1. Inspection of box plots revealed no consistent or extreme outliers in the motor performance data.

**Table 1:** The mean reaction times and accuracy (and their standard deviations) from the explicit motor learning task per block for every stimulation condition (anodal, dual and sham).

		PRE	DURING	POST1	POST2
anodal	RT (*s*)	0.392 (0.134)	0.330 (0.086)	0.315 (0.079)	0.313 (0.076)
	Acc. (*%*)	80.52 (16.99)	80.78 (12.76)	82.84 (11.37)	84.68 (9.57)
dual	RT (*s*)	0.404 (0.111)	0.314 (0.074)	0.303 (0.071)	0.285 (0.063)
	Acc. (*%*)	82.60 (11.67)	82.34 (16.20)	83.35 (9.72)	84.20 (10.58)
sham	RT (*s*)	0.384 (0.076)	0.347 (0.071)	0.336 (0.069)	0.343 (0.069)
	Acc. (*%*)	82.28 (18.02)	82.27 (17.59)	81.39 (21.25)	81.41 (18.13)
The four time points are measurements at baseline (PRE), during tDCS (DURING), immediately after tDCS (POST1) and approximately 15 mins after the end of tDCS (POST2) Reaction times (RT) are expressed in seconds and accuracy (Acc.) in per cent.					

Wrong answers were not excluded in this task, because a missed or a wrong button press still gives insight into the fluidity of finger coordination in repeating the numerical sequence. There was very little fluctuation of accuracy across stimulation condition and thus incorrect answers are evenly distributed across blocks: there was no main effect of time (*F*_*3,57*_ *=0.5, p>0.05; partialη* ^*2*^ *= 0.02*), no main effect of stimulation condition (*F*_*2,38*_ *=0.07, p>0.05; partialη* ^*2*^ *< 0.01*) and no significant interaction (*F*_*6,114*_ *=0.5, p>0.05; partialη* ^*2*^ *= 0.02*). 

There was a significant overall decrease of RTs over time (ANOVA main effect of time *F*_*3,38*_ *=131.57, p<0.001; partialη* ^*2*^ *= 0.87*), but no main effect of stimulation condition (*F*_*2,38*_ *=1.51, p>0.05; partialη* ^*2*^ *= 0.07*). However, there was a significant interaction between time and stimulation condition (*F*_*6,114*_ *=6.624, p<0.001; partialη* ^*2*^ *= 0.26*), suggesting that learning rates varied between stimulation conditions. Subsequent planned ANOVAs were performed to individually contrast each stimulation condition. These contrasts revealed that the differences lay between the dual and the sham condition at the time points DURING (*F*_*1,19*_ *=11.62, p<0.01; partialη* ^*2*^ *= 0.38*), POST1 (*F*_*1,19*_ *=9.65, p<0.01; partialη* ^*2*^ *= 0.34*) and POST2 (*F*_*1,19*_ *=28.86, p<0.001; partialη* ^*2*^ *= 0.60*) when compared to baseline (PRE) as well as at DURING (*F*_*1,19*_ *=11.66, p<0.01; partialη* ^*2*^ *= 0.38*) and POST1 (*F*_*1,19*_ *=12.65, p<0.01; partialη* ^*2*^ *= 0.40*) when compared to POST2. This means that the reduction of RTs by 23% in the dual condition during stimulation was significantly different to the reduction of 9% in the sham condition, with this effect increasing to 30% at POST2 compared with an 11% reduction in RTs in the sham condition at POST2.

The changes in motor task performance in the anodal condition were significantly different to sham condition at POST2 when compared to baseline (F1,19=6.38, p<0.05; *partialη* ^*2*^ *= 0.25*). All other time comparisons revealed non-significant results. This means that only the final performance measurement in the anodal condition was significantly different to baseline with a reduction of RTs by 20% when compared to the sham condition. The anodal and dual conditions were significantly different at POST2 when compared to baseline (*F*_*1,19*_ *=5.64, p<0.05; partialη* ^*2*^ *= 0.23*), but not at any of the levels (all *F*_*1,19*_ *<2.8, p>0.05*; except at the level POST1 vs. POST2 where *F*_*1,19*_ *=3.88, p=0.06; partialη* ^*2*^ *= 0.17*)*.*

In order to control for the practice effects associated with the motor task, the order of conditions every subject underwent was entered as a covariate into the additional ANCOVA. There was no significant interaction between the condition order and the main effect of time (*F*_*3,54*_ *=1.84, p>0.05; partialη* ^*2*^ *= 0.09*) at any of the levels of the variables. There was, however, a significant interaction between condition order and the main effect of stimulation condition (*F*_*6,108*_ *=4.26, p<0.05; partialη* ^*2*^ *= 0.19*) and the planned contrasts revealed that they significantly interact at the level anodal vs. sham (*F*_*1,18*_ *=13.98, p<0.01; partialη* ^*2*^ *= 0.44*). Because of this, the anodal condition was no longer significantly different to sham at any of the time point comparisons, except with near-significance at POST1 when compared to baseline (*F*_*1,18*_=3.91, p=0.06; *partialη* ^*2*^ *= 0.18*). The anodal and dual condition were not significantly different at any of the levels (all *F*_*1,18*_ *<2.1, p>0.05; partialη* ^*2*^ *< 0.1*). After removing the effect of condition order, the dual condition is still significantly different from the sham condition, but due to the stricter experimental control from the ANCOVA on fewer levels than in the ANOVA: dual and sham were significantly different at DURING (*F*_*1,18*_ *=8.82, p<0.01; partialη* ^*2*^ *= 0.33*), POST1 (*F*_*1,18*_ *=5.638, p<0.05; partialη* ^*2*^ *= 0.24*) and POST2 (*F*_*1,18*_ *=10, p<0.01; partialη* ^*2*^ *= 0.36*) when compared to baseline. There was no significant 3-way interaction between condition order, time and stimulation condition at any of the levels of the variables (*F*_*6,108*_ *=0.49, p<0.05; partialη* ^*2*^ *= 0.02*).

 Overall, all subjects improved their finger-coordination in this task over time. The anodal and the dual stimulation conditions improved motor performance further, but this occured earlier and lasted longer in the dual tDCS stimulation condition relative to sham. Performance improvements in the anodal condition were, to some extent, driven by the order that the conditions were administered. There was very little fluctuation of accuracy across the blocks, which means that subjects improved at the task without a noticeable speed/accuracy trade-off.

There was no significant difference in the self-rated visual analogue scale for fatigue, concentration and perceived pain (all p>0.05; all *partialη* ^*2*^ *< 0.02*).

### Neurophysiological Data: Changes in Motor Cortical Excitability

**Figure 4:**
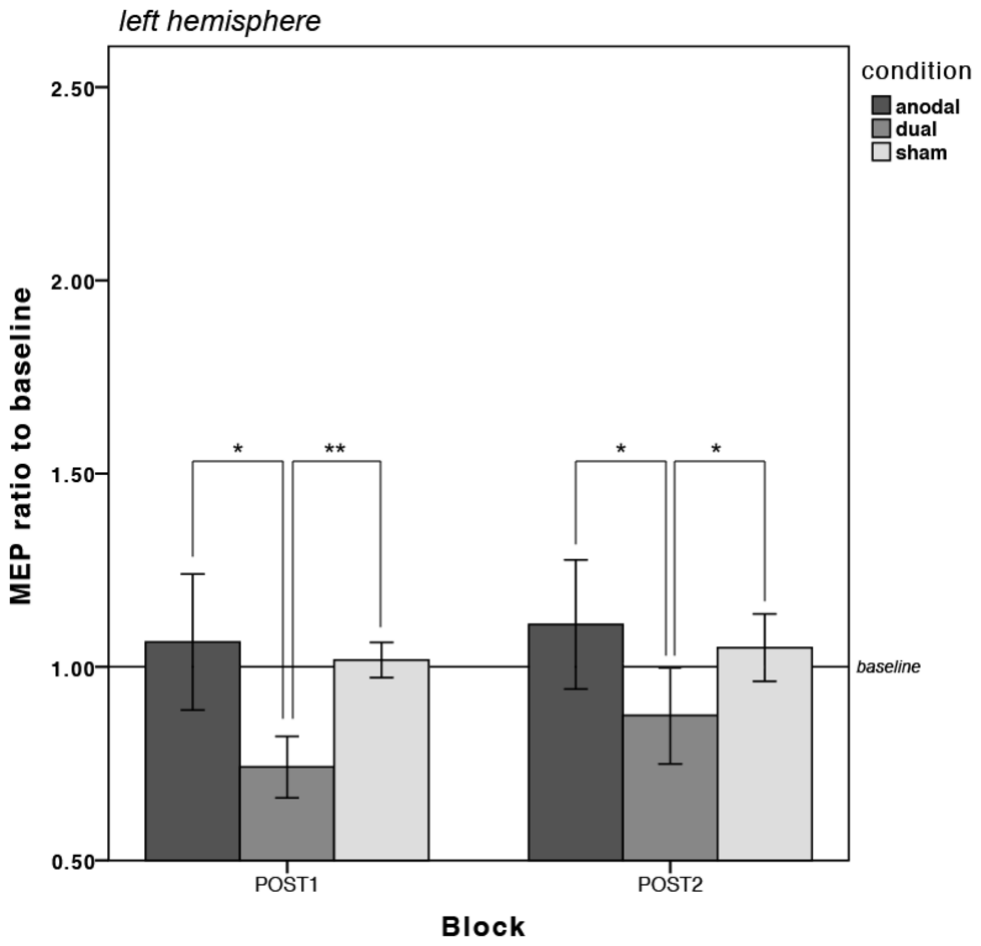
Changes in motor cortical excitability in the LEFT hemisphere for each stimulation condition (anodal, dual and sham). There was a significant reduction in MEPs in the dual condition in the left hemisphere. **=p<0.01, *=p<0.05; Error bars are 95% confidence intervals (CI) calculated on the transformed data and transformed back to the original scale.

**Figure 5:**
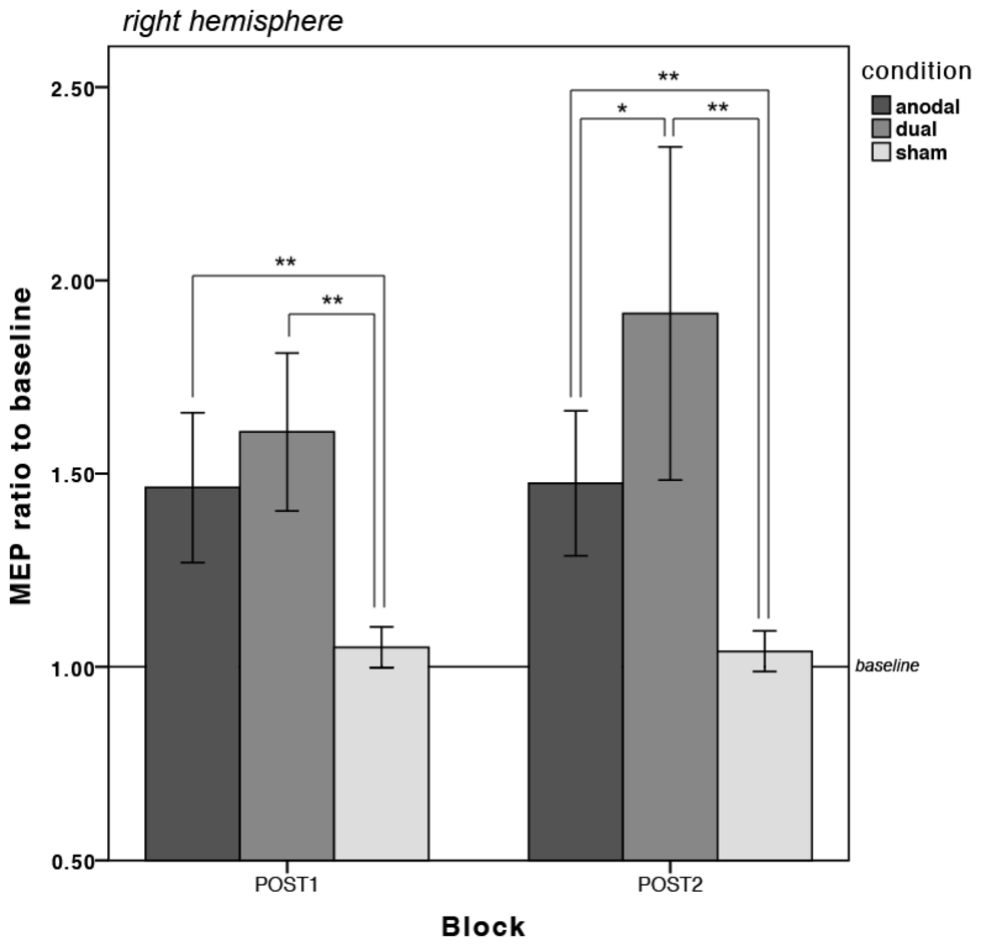
Changes in motor cortical excitability in the RIGHT hemisphere for each stimulation condition (anodal, dual and sham). Both active stimulation conditions were associated with an excitability-enhancing effect at both time points in the right hemisphere, but this was significantly larger in the dual condition. **=p<0.01, *=p<0.05; Error bars are 95% confidence intervals (CI) calculated on the transformed data and transformed back to the original scale.

Table *2* illustrates the mean MEP amplitudes from every measurement per condition and hemisphere and Figure *4* and Figure *5* depict the changes in MEP amplitudes as divided by baseline for the left and right hemisphere respectively. Statistical analyses were carried out separately for the left and right hemisphere. Inspection of box plots revealed no consistent or extreme outliers in the MEP data.

**Table 2:** The average MEP amplitudes (in mV) and their standard deviations per stimulation condition (anodal, dual and sham) and hemisphere (left and right).

		PRE- left	POST1- left	POST2- left	PRE- right	POST1- right	POST2- right
anodal	avg.	0.387	0.415	0.440	0.341	0.486	0.497
	*s.d.*	*0.188*	*0.265*	*0.284*	*0.174*	*0.276*	*0.292*
dual	avg.	0.410	0.308	0.363	0.375	0.563	0.634
	*s.d.*	*0.222*	*0.215*	*0.269*	*0.207*	*0.289*	*0.330*
sham	avg.	0.367	0.372	0.378	0.410	0.434	0.425
	*s.d.*	*0.208*	*0.207*	*0.203*	*0.228*	*0.245*	*0.238*

The four time points are measurements at baseline (PRE), approximately 8 mins after the end of tDCS (POST1) and approximately 20 mins after the end of tDCS (POST2)

For the left hemisphere, these revealed no main effect of stimulation condition (*F*_*2,38*_ *=1.04, p>0.05; partialη* ^*2*^ *= 0.05*) and no main effect of time (*F*_*2,38*_ *=2.31, p>0.05; partialη* ^*2*^ *= 0.11*). There was a significant interaction between the stimulation condition and time (*F*_*4,76*_ *=5.685, p<0.01; partialη* ^*2*^ *= 0.23*). Subsequent planned ANOVAs were performed and contrasting the anodal with the dual condition showed that the mean MEP amplitudes from the dual condition were significantly lower than those from the anodal condition by 31% at POST1 (*F*_*1,19*_ *=6.92, p<0.05; partialη* ^*2*^ *= 0.27*) and by 22% at POST2 (*F*_*1,19*_ *=8.25, p<0.01; partialη* ^*2*^ *= 0.30*) when compared to baseline (PRE), but not when compared to each other (POST1 vs. POST2: *F*_*1,19*_ *=1.18, p>0.05; partialη* ^*2*^ *= 0.06*). The measured MEP in the dual condition were also considerably lower than the mean amplitudes measured in the sham condition by 27% at POST1 (*F*_*1,19*_ *=22.73, p<0.001; partialη* ^*2*^ *= 0.55*) and by 17% at POST2 (*F*_*1,19*_ *=8, p<0.01; partialη* ^*2*^ *= 0.30*) when compared to baseline (PRE).

For the right hemisphere, there was no significant main effect of stimulation condition (*F*_*2,38*_ *=2.35, p>0.05; partialη* ^*2*^ *= 0.11*), however, directly contrasting each level of the variables revealed that the dual condition was significantly different from the sham condition (*F*_*1,19*_ *=5.21, p<0.05; partialη* ^*2*^ *= 0.22*), which means that motor cortical excitability was, overall, significantly higher in the dual than in the sham condition. The main effect of time was significant (*F*_*2,38*_ *=35.6, p<0.001; partialη* ^*2*^ *= 0.65*), which was circumscribed to differences in the levels POST1 (*F*_*1,19*_ *=51.77, p<0.001; partialη* ^*2*^ *= 0.73*) and POST2 (*F*_*1,19*_ *=42.8, p<0.001; partialη* ^*2*^ *= 0.69*) as compared to baseline (PRE). Following a significant interaction of stimulation condition and time (*F*_*4,76*_ *=10.38, p<0.001; partialη* ^*2*^ *= 0.35*), further comparisons showed that mean MEP amplitudes from the dual condition were significantly higher than those from sham by 53% at POST1 (*F*_*1,19*_ *=18.22, p<0.001; partialη* ^*2*^ *= 0.49*) and by 83% at POST2: *F*_*1,19*_ *=25.77, p<0.001; partialη* ^*2*^ *= 0.58*) when compared to baseline. The increase of MEPs by 12% in the dual condition from POST1 to POST2 was also found to be significantly different to sham (*F*_*1,19*_ *=4.5, p<0.05; partialη* ^*2*^ *= 0.19*). Contrasting the anodal and the dual condition revealed significantly higher average MEP amplitudes in the dual condition by 29% at POST2 (*F*_*1,19*_ *=4.66, p<0.05; partialη* ^*2*^ *= 0.2*) but not at POST1 (*F*_*1,19*_ *=1.1, p>0.05; partialη* ^*2*^ *= 0.5*) as compared to baseline and considerably higher average MEP amplitudes in the dual condition at POST1 vs. POST2 with near-significance (*F*_*1,19*_ *=4.2, p=0.055; partialη* ^*2*^ *= 0.18*). When compared to sham, the MEP amplitudes measured in the right hemisphere were significantly higher in the anodal condition than those from the sham condition by 39% at POST1 (*F*_*1,19*_ *=9.6, p<0.01; partialη* ^*2*^ *= 0.34*) and by 41% at POST2 (*F*_*1,19*_ *=10.38, p<0.01; partialη* ^*2*^ *= 0.34*) as compared to baseline. 

While only the dual condition was found to be statistically different to the sham and anodal conditions for the left hemisphere in the form of reduced MEP amplitudes (see Figure *4*), both active conditions were associated with significantly increased MEP amplitudes relative to sham at both time points in the right hemisphere, which was significantly higher in the dual than the anodal condition at POST2 (see Figure *5*).

## Discussion

 We investigated the effects of administering dual tDCS during an explicit motor learning task. The impact tDCS has on motor learning and how this effect is enhanced through task-concurrent stimulation has so far only been demonstrated for the conventional unilateral electrode montage. Our results suggest that simultaneously stimulating both motor cortices further enhances the benefits of task-concurrent stimulation. 

 Both active tDCS conditions showed an improvement in task performance over time, but only the dual condition was found to be significantly different from the sham condition after controlling for the order of conditions. As early as at the time of stimulation onset, reaction times (RTs) were significantly reduced by 23% in the dual condition compared with a 9% reduction in the sham condition (p<0.01). This RT reduction increased to 30% at the final measurement, compared with an 11% reduction in the sham condition at the same time point. There was a clear trend for an effect in the anodal condition (RTs were 20% lower at the last measurement), but this did not reach significance compared to sham (see Figure *2*). Previous studies have demonstrated an enhancement of motor performance using task-concurrent anodal stimulation, but here we demonstrate that this is a weaker non-significant effect. The authors cannot rule out prefrontal contribution in the anodal condition, but positioning and size of electrodes was in accordance with recommendations [30].

Our results confirm that administering tDCS during an explicit motor learning task is beneficial to performance in healthy subjects. The literature suggests this is above that provided by stimulation prior to task execution [21-26,31]. This study demonstrates that the dual montage enhances the performance benefits of task-concurrent stimulation. For the task used in this study, motor learning occurs due to improved finger coordination and is expressed as reduced reaction times. Interestingly, the fastest blocks were also associated with slightly increased accuracy (see Table *1*), which suggests that it was not a strategic adjustment of response threshold (speed-accuracy trade-off), but that real motor learning took place. 

During motor learning, synaptic efficacy in the motor cortex is enhanced, driven by Hebbian synaptic modifications and mediated by long-term potentiation (LTP) and NMDA-specific glutamate receptor function. There is increasing evidence suggesting that the lasting effects of tDCS also involve LTP-like synaptic mechanisms [32-34] and NMDA-receptor activity [35,36], while the immediate modulatory effects of tDCS are polarity-specific modifications of the resting membrane potential [37]. What is crucial during the application of anodal tDCS is that not only NMDA receptors, but also sodium and calcium channels are modulated [36]. Intracellular calcium levels are important for LTP-induction [38] and might, in turn, enhance motor learning effects. 

A more mechanistic explanation for the timing-dependent interaction has previously been attributed to metaplasticity, which is essentially a regulatory mechanism that prevents destabilisation of the existing cortical network from dynamic cortical changes such as LTP. As outlined by Kuo et al. [25], an excitability-enhancement (and thus synaptic strength modulation) prior to and not during performance of a motor task could potentially defocus this process as opposed to enhancing it. Put simply, metaplasticity would ‘step-in’ to prevent further destabilization. Kuo et al. thus argue in favour of applying anodal tDCS during, and not before, motor behaviour to maximise motor learning [25].

In the present study, the dual M1 electrode montage was associated with early and lasting performance improvement. Notably, the blocks with the most decreased reaction times were also associated with the lowest standard deviations, suggesting a stable effect and low inter-subject variability for this condition (see Table *1*). Such an effect was not as easily discernible in the unilateral anodal stimulation condition, which was associated with only a trendwise improvement when compared to sham. Thus, dual M1 stimulation seems to be more suited to increase the rate of motor learning, supporting the conclusion from Vines et al. [11], but this is the first study to demonstrate that this relatively new montage is also very effective when administered during an explicit motor learning task. 

It has been speculated that the measurable improvement in motor performance associated with dual M1 stimulation is mediated by inhibitory interhemispheric projections. Here, the direct excitability- and performance-enhancing effect of the anodal current is amplified by the reduced inhibitory projections coming from the homologous left M1 due to the positioning of the cathodal return electrode. In other words, the motor cortex of the motor-performing hand is additionally released from the normal amount of inhibition originating from its counterpart in the other hemisphere. Studies reporting an increase in motor skill acquisition in the ipsilateral hand following unilateral cathodal stimulation mechanistically support the role of interhemispheric projections in corticomotor functioning [39,40].

Two separate studies, both using resting-state fMRI to investigate the changes in functional connectivity between the primary motor cortices induced by dual tDCS, report a decrease in interhemispheric projections in the unilateral-anodal and the dual condition, but only the dual condition was associated with increases in intracortical projections [12,14]. In another recent fMRI experiment, cortical activation patterns were measured during short tDCS application and it was found that the dual M1 montage induced higher BOLD activity in the region under the anode compared with the unilateral-anodal montage [41]. Taken together, this supports the notion that changing the position of the cathode return electrode (from the supraorbital region to the other M1) causes additional modulation of interhemispheric interactions, which, in turn, augments intracortical projections in the area under the anode. This suggests the mechanisms underlying the enhancing effects of dual stimulation are due to a complex synergistic process involving other areas of motor control rather than a basic additive effect [12].

Consistent with previous research, we observed a simultaneous modulation of cortical excitability in different directions in the dual stimulation condition. That is, the average MEP amplitudes were found to be statistically lower than sham in the left hemisphere and statistically higher than sham in the right hemisphere. This is in keeping with findings reported by Williams et al. [22], who employed stimulation during hand motor training (Jebsen-Taylor Hand Function Task). Another recent study also reports a simultaneous polarity-specific modulation of cortical excitability, but they found that the unilateral anodal montage produced an excitability increase of 30% of the baseline MEP amplitude, the dual M1 montage produced a slightly less robust increase of only 20% [42]. This is complemented by Kidgell et al. [43], who measured changes in motor cortical excitability over the stimulated right M1 of similar magnitude in both the unilateral anodal and the dual tDCS condition. The clear difference was that these studies did not administer a motor task at the same time, unlike in the present study, in which we found that the increase in cortical excitability under the anode was significantly greater in the dual condition than the increase in the anodal condition (see Figure *5*). 

According to the gating principle of plasticity, the increased neuronal firing rates induced by the motor task are further amplified by the excitability-enhancing transcranial stimulation, resulting in increased postsynaptic strength [44]. What follows then is the speculation that when there is motor activity during stimulation, the amount of current that enters the motor cortex is also increased. In an fMRI study, Kwon & Jang [45] report that cortical activity in the area under the anode was increased when the motor task was applied during tDCS when compared to the motor task alone. In another recent study on 44 healthy young adults, Kim & Ko [46] found that the combination of anodal tDCS (2 mA for 20 mins) with voluntary grip exercise increased motor cortical excitability two-fold when compared with single use of either anodal tDCS or voluntary grip exercise. Referring this back to the present findings, it seems that more of both currents entered the motor cortex due to the concurrent application of the motor task and that dual tDCS caused a stronger and increasing facilitation in cortical excitability can only be attributed to the location of the return electrode and the modulation of interhemispheric interactions.

Looking at Figure *2*, the increase in motor learning in the dual condition shows a trend to be increasing as well, so it would have been interesting to include further measurements at later time points to examine how long this trend lasts. Following the findings from Marquez et al. [47], who looked at motor learning over multiple sessions, there could have been additional motor learning taking place between sessions with this explicit finger-coordination task due to offline memory consolidation. The current study also did not include a unilateral-cathodal condition, because this has previously been found not to influence motor performance [24,31]. Reis & Fritsch suggest that the inhibitory effects of cathodal stimulation may be overcome due to an increase in excitability associated with motor training itself [6]. Stagg et al. [23], however, do report a decrease in the overall performance on the finger-sequencing task following cathodal stimulation. Methodological differences in the studies including the nature of the motor task may explain this discrepancy. Another open question is whether the effects outlined in this study are specific to the hemispheres that were stimulated. We focused on non-dominant hand performance due to the increased susceptibility to motor learning, but it has been shown that there are hemispheric asymmetries in interhemispheric inhibition [48,49]. Further research is thus appropriate here to examine if this effect is stable across hemispheres and handedness [50]. 

In conclusion, this study demonstrates that active tDCS applied concurrently to a finger-sequencing coordination task is effective at improving the rate of learning compared with sham stimulation. The bilateral stimulation of both motor cortices is more appropriate than the conventional unilateral anodal stimulation, as motor learning was significantly greater in this condition compared with sham stimulation. In the dual M1 condition, it was also possible to obtain simultaneous modulation of cortical excitability in different directions in the two motor cortices and, further, excitability in the area under the anode was significantly enhanced in the dual compared to the unilateral anodal stimulation condition. Thus, task-concurrent dual M1 stimulation is very effective at determining the desired neuromodulatory effects of tDCS. Optimising these stimulation parameters has important implications for the rehabilitative approaches that are being developed.
